# Applying spectral fractal dimension index to predict the SPAD value of rice leaves under bacterial blight disease stress

**DOI:** 10.1186/s13007-022-00898-8

**Published:** 2022-05-18

**Authors:** YiFei Cao, Huanliang Xu, Jin Song, Yao Yang, Xiaohui Hu, Korohou Tchalla Wiyao, Zhaoyu Zhai

**Affiliations:** 1 College of Engineering, Nanjing Agricultural University, Nanjing, 210032 Jiangsu China; 2 College of Artificial Intelligence, Nanjing Agricultural University, Nanjing, 210095 Jiangsu China; 3 College of Information Engineering, Jiangxi Vocational College of Mechanical & Electrical Technology, Nanchang, 330013 China

**Keywords:** Hyperspectral, Fractal dimension, SPAD value, Vegetation index, Disease stress, Rice

## Abstract

**Background:**

The chlorophyll content is a vital indicator for reflecting the photosynthesis ability of plants and it plays a significant role in monitoring the general health of plants. Since the chlorophyll content and the soil–plant analysis development (SPAD) value are positively correlated, it is feasible to predict the SPAD value by calculating the vegetation indices (VIs) through hyperspectral images, thereby evaluating the severity of plant diseases. However, current indices simply adopt few wavelengths of the hyperspectral information, which may decrease the prediction accuracy. Besides, few researches explored the applicability of VIs over rice under the bacterial blight disease stress.

**Methods:**

In this study, the SPAD value was predicted by calculating the spectral fractal dimension index (SFDI) from a hyperspectral curve (420 to 950 nm). The correlation between the SPAD value and hyperspectral information was further analyzed for determining the sensitive bands that correspond to different disease levels. In addition, a SPAD prediction model was built upon the combination of selected indices and four machine learning methods.

**Results:**

The results suggested that the SPAD value of rice leaves under different disease levels are sensitive to different wavelengths. Compared with current VIs, a stronger positive correlation was detected between the SPAD value and the SFDI, reaching an average correlation coefficient of 0.8263. For the prediction model, the one built with support vector regression and SFDI achieved the best performance, reaching R^2^, RMSE, and RE at 0.8752, 3.7715, and 7.8614%, respectively.

**Conclusions:**

This work provides an in-depth insight for accurately and robustly predicting the SPAD value of rice leaves under the bacterial blight disease stress, and the SFDI is of great significance for monitoring the chlorophyll content in large-scale fields non-destructively.

**Supplementary Information:**

The online version contains supplementary material available at 10.1186/s13007-022-00898-8.

## Background

During the photosynthesis process, chlorophyll, as a plant pigment, is an important material for absorbing light energy [1]. Chlorophyll content is one of the essential factors that directly affect plant growth, and therefore it can be used to reflect the stress of plants [2–4]. For instance, when the rice bacterial blight (BB) disease spreads, rice leaves usually turn from green to yellow, eventually to brown and white. This change means that the chlorophyll content of rice leaves has decreased, leading to the fact that rice leaves have difficulty in photosynthesis [5, 6]. Previous studies have demonstrated that the chlorophyll content of infected rice leaves is negatively correlated with the severity of bacterial blight disease [7, 8]. As a result, timely and accurately evaluating the chlorophyll content of rice leaves is an efficient measure for monitoring the rice BB disease.

At present, the soil–plant analysis development (SPAD) meter is widely adopted to evaluate the chlorophyll content due to its advantages like low-cost, ease of use, nondestructive testing, etc. [9]. Various publications have reported that the SPAD value and the chlorophyll content are positively correlated [10, 11]. Therefore, it is possible to directly measure the SPAD value for monitoring the general health of plants instead of conducting chlorophyll measurements through laboratory tests. However, hand-held SPAD instruments are not suitable for measurements in large-scale fields [12].

To overcome this scalability problem, researchers are now applying hyperspectral sensors to the agricultural domain [13, 14], thanks to the rapid development of the remote sensing technique. For evaluating the chlorophyll content, measuring the SPAD value by hyperspectral images has drawn worldwide attention and achieved great success [15–18]. Zhang [19] combined multiple wavelength regions to define a novel index for evaluating the relative chlorophyll content in sugar beet. The performance of this index was verified through a 3-year field experiment, achieving the best prediction accuracy of SPAD value with a coefficient of determination (R^2^) of 0.83. Yoshitomo [20] designed a hyperspectral sensing system to estimate the SPAD value by the normalized difference vegetation index (NDVI). The results showed that the correlation coefficient between the NDVI and the SPAD value reached 0.85 at night and 0.77 in the day.

From above works, it is detected that various vegetation indices (VIs) have been adopted to evaluate the chlorophyll content of plants. Although these VIs could achieve promising results, most of them are usually calculated based on several visible spectra like red, green, and blue edge regions. It is noted that some wavelength regions are not considered in current VIs. The ignorance of certain regions may result in the loss of spectral information related to the chlorophyll content evaluation, thereby decreasing the prediction accuracy [21, 22]. Meanwhile, a few studies have contributed to the assessment of chlorophyll content in plant leaves under disease stress. As the disease condition becomes worse, the chloroplast and cell structure in plant leaves would be further damaged. When the chlorophyll content is reduced, the SPAD value would decrease as well, and even exceed the estimated range of current VIs [6, 23]. It is also worth noting that the reflectance of visible spectra would increase when plants are infected by diseases. Furthermore, changes of spectra caused by the geometric configuration, shape, and reflectance would also have an influence on the accuracy of the SPAD value measurement [24, 25]. Conclusively, current VIs cannot fully detect changes of the SPAD value in diseased plant leaves [26]. There is an urgent need to develop new evaluation methods by considering more spectral information to overcome the accuracy issue of SPAD value prediction under disease stress.

As a popular branch in the mathematics domain, fractal dimension is potentially applicable to analyze the spectral information [27]. A fractal dimension can be seen as a statistical index that characterizes patterns by quantifying the irregularity as a ratio of the change in detail to the change in scale. The major benefit of the fractal dimension is its sensitivity to changes of the spectral information, caused by the geometric configuration, shape, reflectance, etc. [28]. These changes are the main factors that reflect the general health of plants [29, 30]. By calculating the fractal dimension over the spectrum of infected leaves, adequate spectral information could be obtained. Under this circumstance, the evaluation of the SPAD value depends on the use of all spectral bands instead of selected ones, which may improve the evaluation accuracy [31]. Moreover, the fractal dimension is also able to quantify the irregularity of the spectrum due to its advantageous nature [28, 32, 33].

In this work, we applied the fractal dimension to analyze the hyperspectral information to predict the SPAD value of rice leaves under the BB disease stress. A novel vegetation index, namely the spectral fractal dimension index (SFDI), is proposed. After comparing four machine learning models, including support vector regression (SVR), decision tree (DT), partial least squares regression (PLSR), and back propagation neural network (BPNN), we selected the optimal one and combined SFDI with it to establish a prediction model to evaluate the SPAD value of rice leaves under the bacterial blight disease stress. This work demonstrates that compared with current VIs like MSAVI, NDVI, PRI, and so on, the proposed SFDI can more accurately predict the SPAD value of rice leaves under different bacterial blight disease levels.

## Materials and methods

### Experimental design

A moderately resistant rice cultivar, namely “Nanjing 9108”, was selected as the experimental material. After soaking and germinating, we planted the seeds in a facility environment with an average temperature of 30 °C and an average air humidity of 75%. To lower the planting density, emerged rice was transplanted to individual pots, and each pot contained 3 rice plants. In total, 50 healthy plants were chosen to be infected with *Xanthomonas oryzae pv. oryzae* (Additional file 1: Fig. S1a). For the purpose of infection, we used a pair of scissors dipped in pathogenic bacteria to cut off the top of a healthy rice leaf when the 5th leaf of rice emerged (Additional file 1: Fig. S1b).

For obtaining the hyperspectral image, we adopted a push broom hyperspectral imaging system (Isuzu Optics Corp, Taiwan, China). The main components of this imaging system and their parameters are listed in Additional file 1: Table S1. The system software is composed of Spectral-image and the HSI Analyzer. Totally, for a single rice leaf, the reflectance of 306 bands is obtained, ranging from 378 to 1033 nm.

**Fig. 1:**
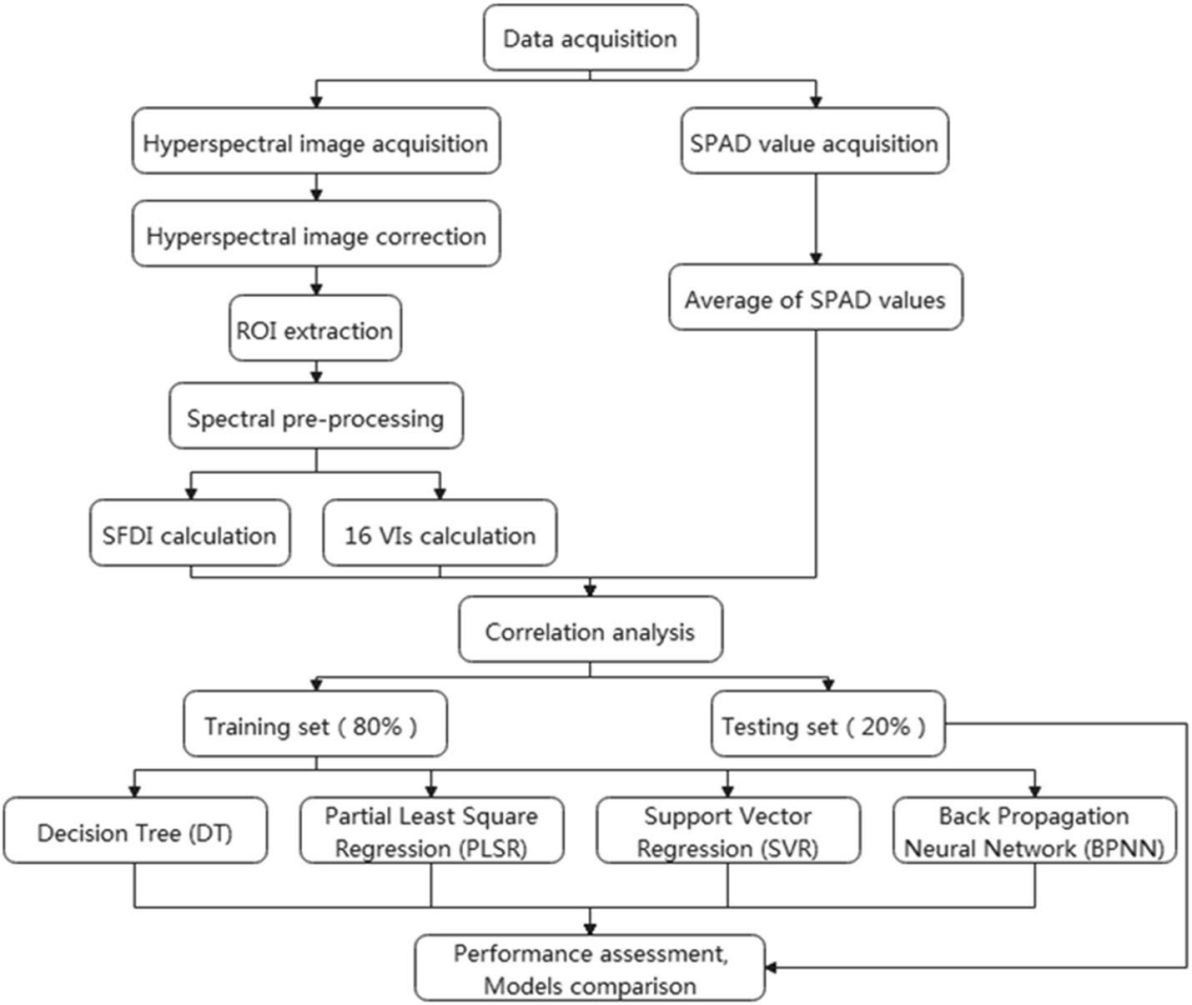
Workflow of predicting the SPAD value of rice leaves under BB disease stress

In general, the workflow of predicting the SPAD value of rice leaves under the bacterial blight disease is illustrated in Fig. 1. First, we preformed pre-processing operations on the collected raw spectra, including HSI correction and smoothing. Then, the SFDI and selected VIs were calculated. We also used the SPAD meter to measure the SPAD value as the ground truth. The correlation between all vegetation indices and the SPAD value was then analyzed. Lastly, the performance of machine learning based SPAD value prediction models was assessed.

### Hyperspectral image acquisition and pre-processing

#### Hyperspectral image acquisition

Before imaging, the light source was turned on and preheated for 5 min to produce a stable light source. The imaging parameters for collecting the spectra in this experiment are presented in the Additional file 1: Table S2.

**Fig. 2:**
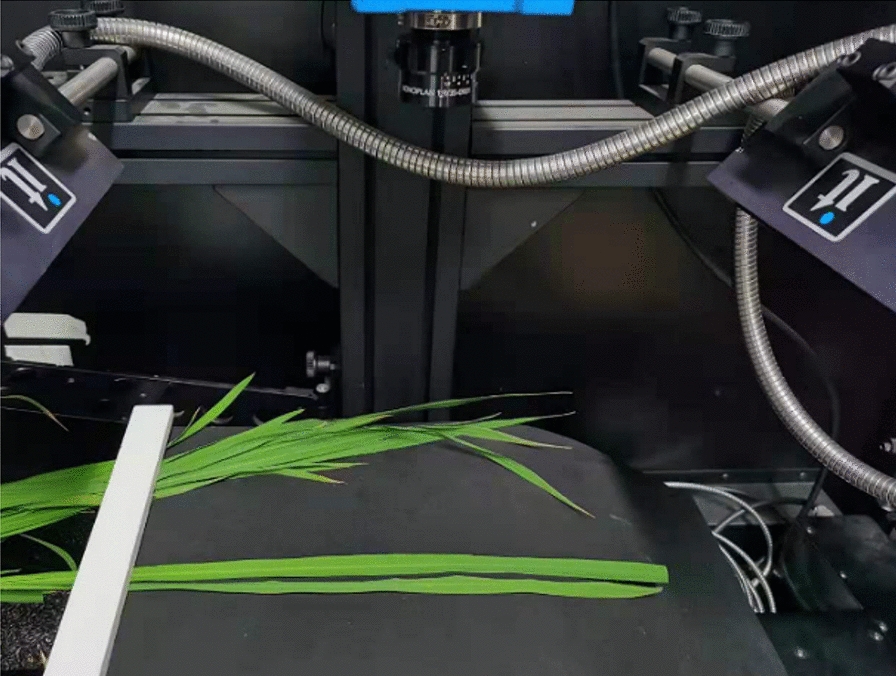
Display diagram of the hyperspectral imaging system

In order to eliminate the background noise, rice leaves were fixed on a black cardboard before imaging. Next, the black cardboard is placed on a shifting platform. The duration of measurement lasts for 3 weeks. Every other day, we measured two leaves of each sample of rice plant, and the HSI of selected leaves was collected 10 times. Finally, we obtained 500 hyperspectral images in total. The imaging system is shown in Fig. 2.

Considering that the moving speed of the shifting platform and the exposure duration may have an influence on the resolution of images and cause distortion, we calibrated the obtained images by following Eq. (1) to reduce noise and to improve the stability and accuracy of further analysis.

1 $$ R(i) = \frac{{I_{r} (i) - I_{d} (i)}}{{I_{w} (i) - I_{d} (i)}}, $$

where $R(i)$ denotes the reflectance, $I_{r} (i)$ denotes the uncalibrated reflectance of the obtained images, $I_{w} (i)$ denotes the reflectance of a white panel, and $I_{d} (i)$ denotes the substitute for the dark current and noise when the camera shutter is closed.

#### Disease level categorization

After segmenting the obtained 500 original images, we built a dataset with 1000 hyperspectral images and each image only contained a single rice leaf. According to the GB/T 17980.19-2000 standard (http://www.gbstandards.org/GB_standard_english.asp?code=GB/T%2017980.19-2000) presented in Table 1, the bacterial blight disease level can be categorized into six classes by visual inspection.

**Table 1:** Categorization standard of rice leaves under the bacterial blight disease

Disease level	Symptoms
Level 0	No clear spot is shown
Level 1	It appears 2–3 cm white spots, or even few brown spots are shown. The spot area is account for 10% of the leaf
Level 2	The length of appeared spots is less than a quarter of the leaf’s length, or the spot area is account for 20% of the leaf
Level 3	The length of appeared spots is between a quarter and half of the leaf’s length, or the spot area is account for 20–49% of the leaf
Level 4	The length of appeared spots is between a half and three quarters of the leaf’s length, or the spot area is account for 50–74% of the leaf
Level 5	The length of appeared spots reaches beyond three quarters of the leaf’s length, or the spot area is account for more than 75% of the leaf

**Fig. 3:**
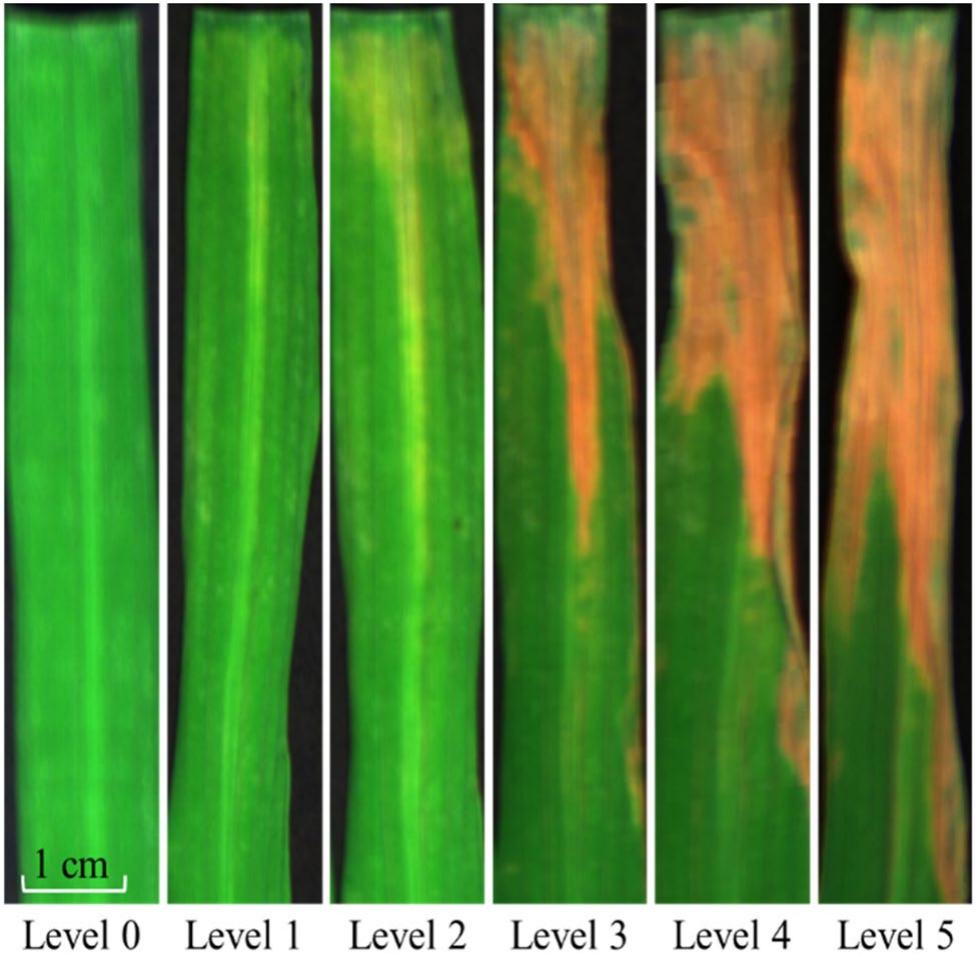
Leaves under different disease levels

In the dataset, the number of rice leaf hyperspectral images labelled by level 0 to 5 is 200, 170, 160, 200, 140, and 130, respectively. An example of leaf images with different labels is displayed in Fig. 3.

#### Hyperspectral image pre-processing

To reduce the noise caused by the imaging system and environmental factors, hyperspectral images were synthesized by the HSI Analyzer. After extracting the region of interest (ROI) through ENVI 5.1x (Research System Inc., Boulder, CO., USA), we smoothed the original spectrum and calculated the average spectrum. The size of the ROI in this work is 50 × 50 pixels.

The smoothing operation is widely used to eliminate the interference of high-frequency noise in raw spectral data and to improve the spectral signal-to-noise ratio. The Savitzky–Golay (SG) algorithm is a popular method to smooth the raw spectrum by calculating the average value of a set of sample raw spectra following a moving smoothing window [34]. Since various researches have reported its effectiveness [35], we adopted the SG algorithm in this work as well (Additional file 1: Fig. S2). The SG smoothing filter has a kernel size of 5 × 5 × 5 and the polynomial order is 3. The filter calculates the filtered value at the central node of the kernel. The SG convolution uses the least square fitting coefficient as the digital filter response function to perform convolutional smoothing.

It is noted that both ends (wavelength less than 420 nm and greater than 950 nm) of the spectrum contain much noise. Considering the number of subsequent data and computation costs, Pi [36] removed the less informative and noisy 40 bands at both ends of the spectrum. In this work, the noise at both ends of the original spectrum is also high. Therefore, we removed both ends of the original spectrum and the wavelength ranging from 420 to 950 nm was maintained for further analysis.

### SPAD value acquisition

Many studies have demonstrated that the SPAD value and the chlorophyll content are positively correlated [37]. The change of the SPAD value can reflect the change of the chlorophyll content accordingly [38]. In this work, for ensuring the correctness of hyperspectral analysis, we used the SPAD-502 meter to directly measure the SPAD value of rice leaves under the bacterial blight disease stress.

A hand-held SPAD-502 meter, produced by KONICA MINOLTA (JAPAN), was used in the experiment (Additional file 1: Fig. S3, Table S3). The SPAD-502 meter can determine the chlorophyll concentration by measuring the leaf absorbance in red-light and near-infrared regions. Two LEDs with peak wavelengths of 650 and 940 nm emit the light. After the light passes through the sample leaf in the measuring head, the receptor will count the amount of passed light and convert it into electrical signals, thereby calculating the SPAD value and displaying the result on the screen in real time [11].

**Fig. 4:**
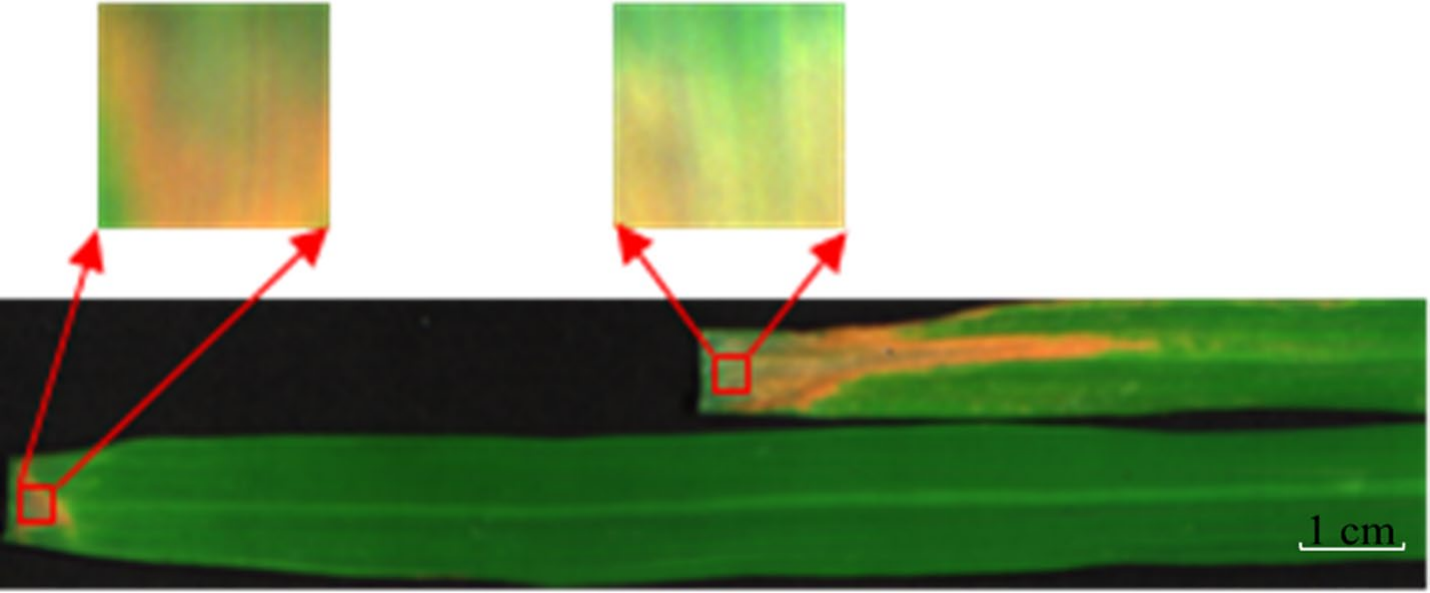
ROI selection diagram. The size of the ROI is 50 × 50 pixels

To ensure that the rice leaves are infected with the bacterial blight disease successfully, we inoculated leaves by artificially cutting them at their tips. In this way, the pathogen can spread along the leaf vascular tissue and infect rice leaves. The stress symptom at the tip of the leaf was more obvious, so we selected the ROI from the tip for investigation. Considering the deviation caused by the difference of leaf thickness, we chose the sampling position near the cutoff point as the ROI (50 × 50 pixels), as shown in Fig. 4. In our experiment, because the ROI was small, for a single sample, we measured the SPAD value three times and calculated the average value for record. Finally, we obtained 1000 SPAD values and categorized them under six disease levels.

### Spectral fractal dimension index calculation

**Fig. 5:**
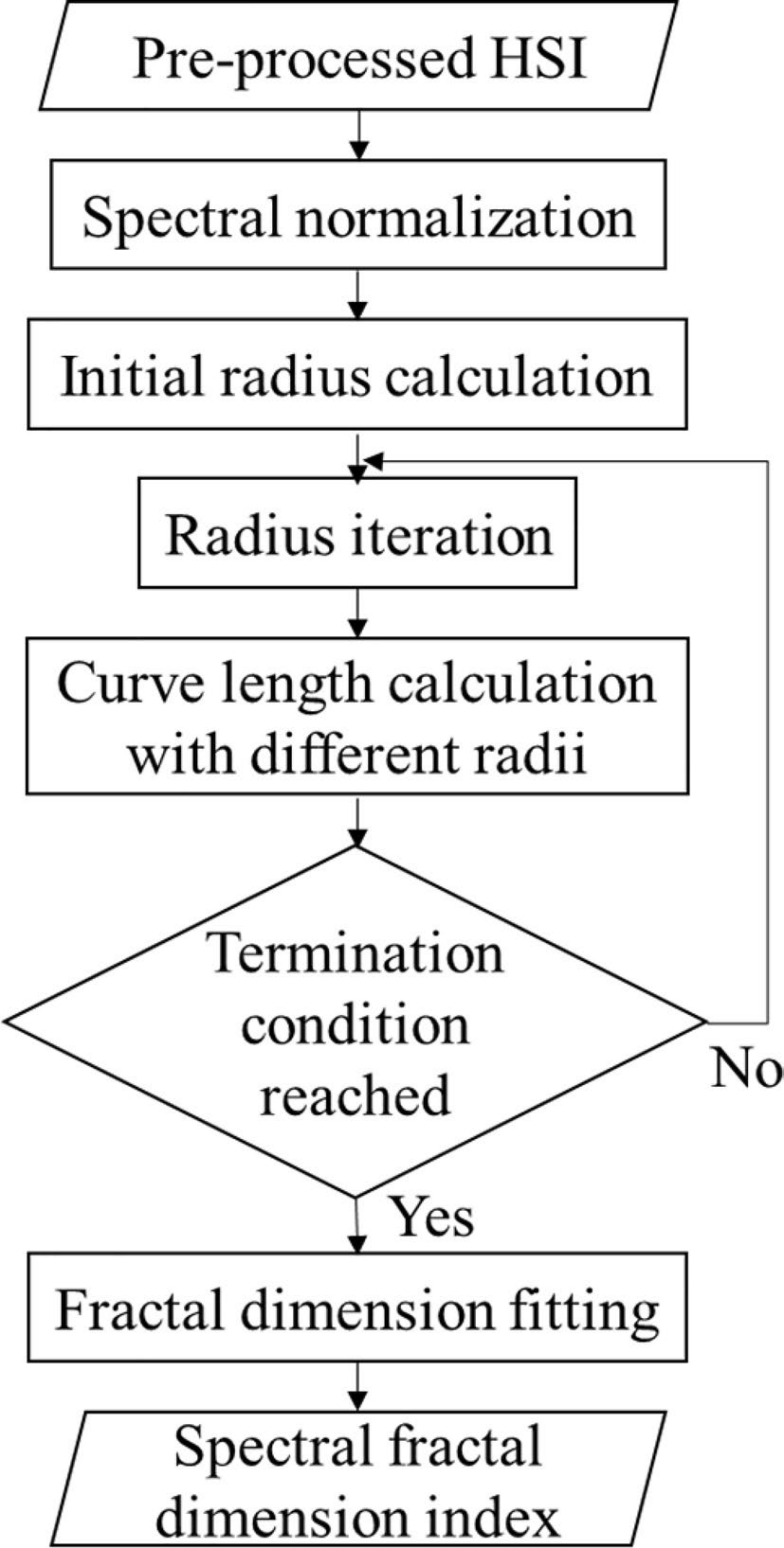
Flow chart of computing the spectral fractal dimension index

As a matter of fact, the reflectance curve in any pixel area of a hyperspectral image can be represented by a trademark shape [39, 40]. However, this shape is considered as an irregular curve, which cannot be characterized by any numerical equation. Here, the fractal geometry is introduced. As a branch of the mathematics domain, fractal dimension is good at describing shapes, especially irregular ones. The fractal dimension of any curve can be measured by its irregularity, thereby being treated as its characteristic feature [28]. It is worth mentioning that the fractal dimension can provide a comprehensive description of spatial curves. For the hyperspectral data, if the spectral signals of pixels are plotted against bands, they will generate a corresponding hyperspectral curve [41]. In general, the fractal dimension can be computed by the box dimension method, the variance method, the structure function method, and so on [29]. In this work, we computed the fractal dimension by iterating the radius. The details of computing the proposed SFDI are presented in Fig. 5, following three main steps.

1. Spectral normalization
  The reflectance of calibrated hyperspectral curve ranges from 0 to 1, therefore, we adopted the maximum and minimum normalization method to eliminate the influence of wavelength magnitudes. The mathematical model of wavelength normalization is formulated in Eqs. (2) and (3) as follows.
  2 $$ \upvarphi = \left( {x_{1} ,y_{1} ;x_{2} ,y_{2} ; \ldots ;x_{n} ,y_{n} } \right), $$
  3 $$ x_{i} = \frac{{x - x_{min} }}{{x_{max} - x_{min} }}, $$
  where $\upvarphi$ denotes the curve of the hyperspectral pixel, $x_{n}$ and $y_{n}$ denote the *n*th wavelength of the pre-processed spectrum, $x_{i}$ denotes the wavelength after normalization, $x_{min}$ and $x_{max}$ are the minimum and maximum wavelength values, respectively.
2. Radius iteration
  Radius iteration is the core of computing the fractal dimension. Because the length of hyperspectral curves can be quantified by different radii. The radius in the current iteration can be used to detect minor changes of the hyperspectral reflectance, according to fractal theory. As the radius decreases, the length of the hyperspectral curve would become stable. The radius and the length of the curve are correlated by exponents [40]. We computed the fractal dimension of the average hyperspectral curve for each ROI by following the below sub-steps.
  Sub-step 1: The hyperspectral curve ranging from 420 to 950 nm is read and the total number of bands is counted. The starting and ending coordinates are $\left( {x_{1} ,y_{1} } \right)$ and $\left( {x_{n} ,y_{n} } \right)$, respectively. The initial radius is defined in Eq. (4).
    4 $$ r_{1} = \frac{1}{2}\left( {\frac{1}{n - 1}\mathop \sum \limits_{i = 1}^{n - 1} \sqrt {\left( {x_{i + 1} - x_{i} } \right)^{2} + \left( {y_{i + 1} - y_{i} } \right)^{2} } } \right),\left( {i = 1,2, \ldots ,n - 1} \right), $$
    where $r_{1}$ denotes the initial radius.
  Sub-step 2: Considering the starting coordinate $\left( {x_{1} ,y_{1} } \right)$ as the center of an arc, we drew an arc to intersect the hyperspectral curve. At this time, we obtained an intersection point and treated it as the center of an arc for drawing the next. The drawing of arcs is repeated along the direction of the hyperspectral curve until the distance between the center of the last arc and the ending coordinate $\left( {x_{n} ,y_{n} } \right)$ is less than the initial radius. Here, we can obtain the number of arcs, denotes as $N\left( {r_{1} } \right)$.
  Sub-step 3: For further exploring the minor changes of the hyperspectral curve, we changed the radius to draw the arc. The radius $r_{j}$ is iterated by Eq. (5), while the curve length $L\left( {r_{j} } \right)$ in each iteration with different radii is computed by Eq. (6).
    5 $$ r_{j + 1} = r_{j} /\sqrt 2 ,{ }\left( {j = 1,2, \ldots ,M - 1} \right) , $$
    6 $$ L\left( {r_{j} } \right) = N\left( {r_{j} } \right) \times r_{j} , $$
    where $r_{j}$ denotes the radius of the *j*th iteration, $N\left( {r_{j} } \right)$ denotes the number of arcs drew by $r_{j}$, $L\left( {r_{j} } \right)$ denotes the curve length of the *j*th iteration, $M$ denotes the maximum number of iterations.
  The termination condition of the radius iteration is defined that the curve length in the neighboring iteration is equal or less than $r_{M} /M$, described by Eq. (7).
  7 $$ L\left( {r_{M} } \right) - L\left( {r_{M - 1} } \right) \le \frac{{r_{M} }}{M} $$
  Figure 6 shows the process of the spectral curve length measurement for radius $r_{1}$, $r_{j}$ and $r_{M}$, respectively. It can be seen from Fig. 6 that a smaller radius can more clearly reflect the irregularity of hyperspectral curves. The more arcs are obtained, the more accurate the curve length will be, thereby providing a more robust result [33].

**Fig. 6:**
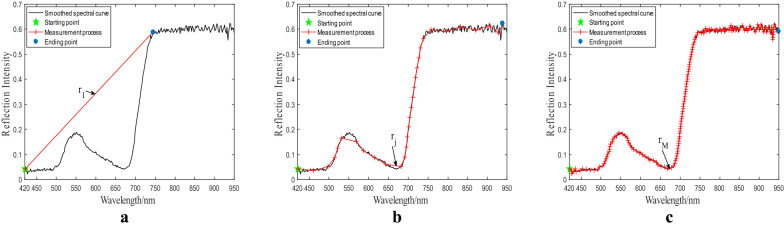
Measurement of the hyperspectral curve length by radius iteration. **a** Measurement with the initial radius r_1_. **b** Measurement with iterated radius r_j_. **c** Measurement the last radius r_M_. After initialization, the radius keeps being updated according to Eq. (5) during iteration. The termination condition is determined by Eq. (7)
3. Fractal dimension logarithmic fitting
  The fractal dimension of 420–950 nm bands, denoted by $F_{D}$, is fitted by the logarithmic of Hausdorff dimension [42] and the formula is defined in Eq. (8).
  8 $$ {\text{F}}_{{\text{D}}} = - \frac{{{\text{lg}}\left( {N\left( {r_{j} } \right)} \right)}}{{{\text{lg}}\left( {1/r_{j} } \right)}},{ }\left( {j = 1,2, \ldots ,M} \right), $$
  The value of the fractal dimension $F_{D}$ is the proposed SFDI and it lies in the interval from 1 to 2.
  The SFDI is a powerful representation to reflect the irregularity of the hyperspectral curve. The selected bands (e.g., visible and near-infrared bands) cover almost all the necessary information for predicting the SPAD value. By iterating the radius, minor changes in the reflectance of neighboring bands can be detected and the relationship between them can be patterned. Thus, the proposed SFDI provides solid support for monitoring the SPAD value of rice leaves under the bacterial blight disease stress.

### Definition and calculation of current VIs

Various researches have demonstrated the strong positive correlation between the visible light, red edge, and near-infrared regions and the SPAD value [43, 44]. In the visible region, the ideal wavelengths for predicting the SPAD value are near 550 and 670 nm, which correspond to the absorption peaks in the red and blue regions [45]. As a result, these two wavelengths are usually used as sensitive indicators to quantify the SPAD value. In addition, the wavelengths near 500 and 750 nm are often treated as anti-interference indicators because these two wavelengths correspond to the absorption valleys [46]. Based on these sensitive and anti-interference indicators, for quantifying the SPAD value, researchers have formulated various VIs like the modified soil-adjusted vegetation index (MSAVI), normalized difference vegetation index (NDVI), photochemical reflectance index (PRI), modified chlorophyll absorption in reflective index (MCARI), etc. Some popular indices are listed in Table 2.

**Table 2:** Definition of current VIs

VIs	Definition or equation	References
GNDVI	$\left( {{\text{R}}_{800} - {\text{R}}_{550} } \right)/\left( {{\text{R}}_{800} + {\text{R}}_{550} } \right)$	[45]
MCARI	$\left[ {\left( {{\text{R}}_{700} - {\text{R}}_{670} } \right) - 0.2\left( {{\text{R}}_{700} - {\text{R}}_{550} } \right)} \right]\left( {{\text{R}}_{700} /{\text{R}}_{670} } \right)$	
PSRI	$\left( {{\text{R}}_{680} - {\text{R}}_{500} } \right)/{\text{R}}_{750}$	[46]
VOG_1_	${\text{R}}_{740} /{\text{R}}_{720}$	[47]
VOG_2_	$\left( {{\text{R}}_{737} - {\text{R}}_{747} } \right)/\left( {{\text{R}}_{715} + {\text{R}}_{726} } \right)$	
VOG_3_	$\left( {{\text{R}}_{737} - {\text{R}}_{747} } \right)/\left( {{\text{R}}_{715} + {\text{R}}_{720} } \right)$	
MSAVI	$\frac{1}{2} \times \left[ {\left( {2{\text{R}}_{{{\text{Nir}}}} + 1} \right) - \sqrt {\left( {2{\text{R}}_{{{\text{Nir}}}} + 1} \right)^{2} - 8\left( {{\text{R}}_{{{\text{Nir}}}} - {\text{R}}_{{{\text{Red}}}} } \right)} } \right]$	[48]
NDVI	$\left( {{\text{R}}_{800} - {\text{R}}_{670} } \right)/\left( {{\text{R}}_{800} + {\text{R}}_{670} } \right)$	[49]
PRI	$\left( {{\text{R}}_{570} - {\text{R}}_{531} } \right)/\left( {{\text{R}}_{570} + {\text{R}}_{531} } \right)$	
NPCI	$\left( {{\text{R}}_{680} - {\text{R}}_{430} } \right)/\left( {{\text{R}}_{680} + {\text{R}}_{430} } \right)$	[50]
MTCI	$\left( {{\text{R}}_{754} - {\text{R}}_{709} } \right)/\left( {{\text{R}}_{709} - {\text{R}}_{681} } \right)$	[51]
RVI	${\text{R}}_{{{\text{Nir}}}} /{\text{R}}_{{{\text{Red}}}}$	[52]
NDI	$\left( {{\text{R}}_{800} - {\text{R}}_{680} } \right)/\left( {{\text{R}}_{800} + {\text{R}}_{680} } \right)$	[53]
SAVI	$1.5 \times \left( {{\text{R}}_{800} - {\text{R}}_{670} } \right)/\left( {{\text{R}}_{800} + {\text{R}}_{670} + 0.5} \right)$	[54]
VARI_green_	$\left( {{\text{R}}_{560} - {\text{R}}_{670} } \right)/\left( {{\text{R}}_{560} + {\text{R}}_{670} - {\text{R}}_{450} } \right)$	[55]
VARI_red_	$\left( {{\text{R}}_{700} - 1.7{\text{R}}_{670} + 0.7{\text{R}}_{450} } \right)/\left( {{\text{R}}_{700} + 2.3{\text{R}}_{670} - 1.3{\text{R}}_{450} } \right)$	

${\text{R}}_{800}$: spectral reflection intensity at 800 nm, the same goes for ${\text{R}}_{754}$, ${\text{R}}_{680}$, ${\text{R}}_{450}$, and so on

${\text{R}}_{{{\text{Nir}}}}$: mean reflection intensity between 760 and 850 nm

${\text{R}}_{{{\text{Red}}}}$: mean reflection intensity between 650 and 670 nm

It is acknowledged that the accuracy of predicting the SPAD value through current VIs would be affected the plant disease stress. Consequently, establishing these VIs with few wavelengths to predict the SPAD value is potentially risky. Although the combination of several wavelengths is used for formulation of VIs, the selected wavelengths cannot fully cover the sensitive information, thereby decreasing the SPAD value prediction accuracy.

### Evaluation criteria

All the hyperspectral information is processed by a computer under the Windows 10 operating environment, with 16 GB RAM and Inter QuadCore i7-8700 (4.2 GHz). The analyzer tool is HSI Analyzer, ENVI5.3 and Matlab R2016a.

The correlation of determination (R^2^), root mean square error (RMSE), and relative error (RE) are selected as the evaluation criteria [56]. As the R^2^ value increases and the values of RMSE and RE decrease, the prediction model performs better. Formulations of evaluation criteria are defined in Eqs. (9–11).

9 $$ {\text{R}}^{2} = 1 - \frac{{\mathop \sum \nolimits_{{{\text{i}} = 1}}^{{\text{n}}} \left( {{\hat{\text{y}}}_{{\text{i}}} - {\text{y}}_{{\text{i}}} } \right)^{2} }}{{\mathop \sum \nolimits_{{{\text{i}} = 1}}^{{\text{n}}} \left( {{\text{y}}_{{\text{i}}} - {\overline{\text{y}}}_{{\text{i}}} } \right)^{2} }}, $$

10 $$ {\text{RMSE}} = \sqrt {\frac{1}{{\text{n}}}\mathop \sum \limits_{{{\text{i}} = 1}}^{{\text{n}}} \left( {{\hat{\text{y}}}_{{\text{i}}} - {\text{y}}_{{\text{i}}} } \right)^{2} } , $$

11 $$ {\text{RE}} = \frac{1}{n}\mathop \sum \limits_{{{\text{i}} = 1}}^{{\text{n}}} \frac{{\left| {{\hat{\text{y}}}_{{\text{i}}} - {\text{y}}_{{\text{i}}} } \right|}}{{{\text{y}}_{{\text{i}}} }} \times 100\% {,} $$

where ${\hat{\text{y}}}_{{\text{i}}}$ denotes the predicted value, ${\text{y}}_{{\text{i}}}$ denotes the ground truth, ${\overline{\text{y}}}_{{\text{i}}}$ denotes the average value of ${\text{y}}_{{\text{i}}}$, and *n* denotes the number of samples.

## Results

### Changes of spectral characteristics of disease leaves

#### Changes of hyperspectral reflectance curve

**Fig. 7:**
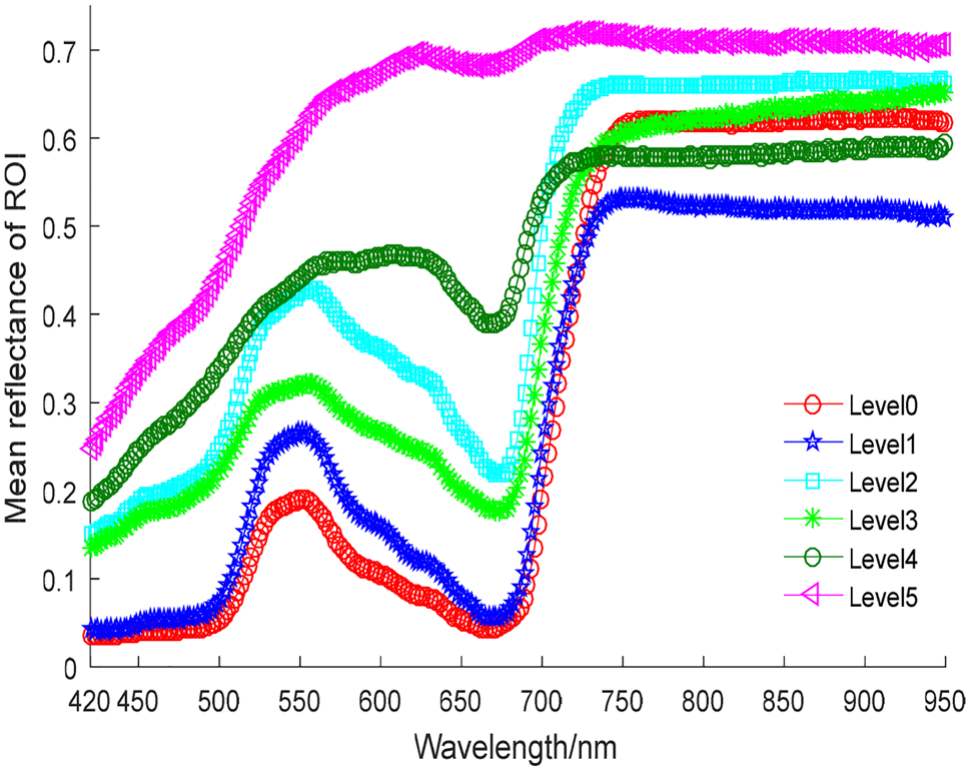
Mean hyperspectral curves of ROI from a single leave under six disease levels. For illustrating the changes of the hyperspectral reflectance, we monitored the reflectance of a single leaf after infection (when the severity is Level 0). The severity of the bacterial blight disease develops with time, until it reaches to Level 5

Rice infected by the bacterial blight disease will experience a decrease in chlorophyll synthesis and photosynthesis. Furthermore, the hyperspectral reflectance of infected rice leaves will change as well. In infected rice leaves, the hyperspectral curve varied in disease levels. The mean hyperspectral curves of this selected leaf under six disease levels are shown in Fig. 7.

Dark green water-stained streaks would appear in the infected region of rice leaves during the early stages of bacterial blight infection. As the BB disease develops further, the infected leaves turn gray-green and become curled. The reflectance in the visible region is increased, especially in the red valley region (between 650 and 680 nm) increases sharply, the increasing rate is much higher than that of the green peak regions (525 to 575 nm). Until diseased rice leaves turn yellow and white, the highest reflectance is reached. Due to the hyperspectral characteristics of the visible light region are mainly affected by the absorption of the pigment, the increasing rate of reflectance in the visible region is higher than that in the near-infrared region, which is supported by An [57]. It is concluded that the hyperspectral reflectance of rice leaves changes with the SPAD value after the bacterial blight infection, which provides a theoretical basis for the next step in analyzing the correlation between the SPAD value and the hyperspectral reflectance.

### SFDI and SPAD variation analysis

From the above analysis, it can be seen that the hyperspectral reflectance of rice leaves under different disease levels has major differences, and the irregularity of hyperspectral curves is distinguishable. The average, maximum, and minimum of the proposed SFDI under six different disease levels are shown in Table 3.

**Table 3:** Statistical data of SFDI under different disease levels

Disease level	Mean	Maximum	Minimum
Level 0	1.1807	1.2042	1.1372
Level 1	1.2190	1.2408	1.1975
Level 2	1.2595	1.2895	1.2401
Level 3	1.2779	1.3036	1.2483
Level 4	1.3199	1.4126	1.2627
Level 5	1.3962	1.5040	1.3595

It can be seen from Table 3 that the SFDI values under different disease levels have major differences. As the bacterial blight disease develops, the mean, maximum, and maximum values of SFDI are eventually increased.

Influenced by the cell structure and leaf chlorophyll content, there are large reflective surface cavities within the spongy tissue structure of healthy rice leaf flesh, and the intracellular chlorophyll is in a hydrosol state, leading to strong infrared reflection and a large SPAD value. For rice leaves under the bacterial blight disease stress, after the bacterial blight disease invaded leaves, the leaf chloroplasts and structure are damaged, which would hinder the synthesis of the leaf chlorophyll and the smooth progress of photosynthesis. The chlorophyll content of rice leaves gradually decreases, and the SPAD value would also decrease [38].

**Fig. 8:**
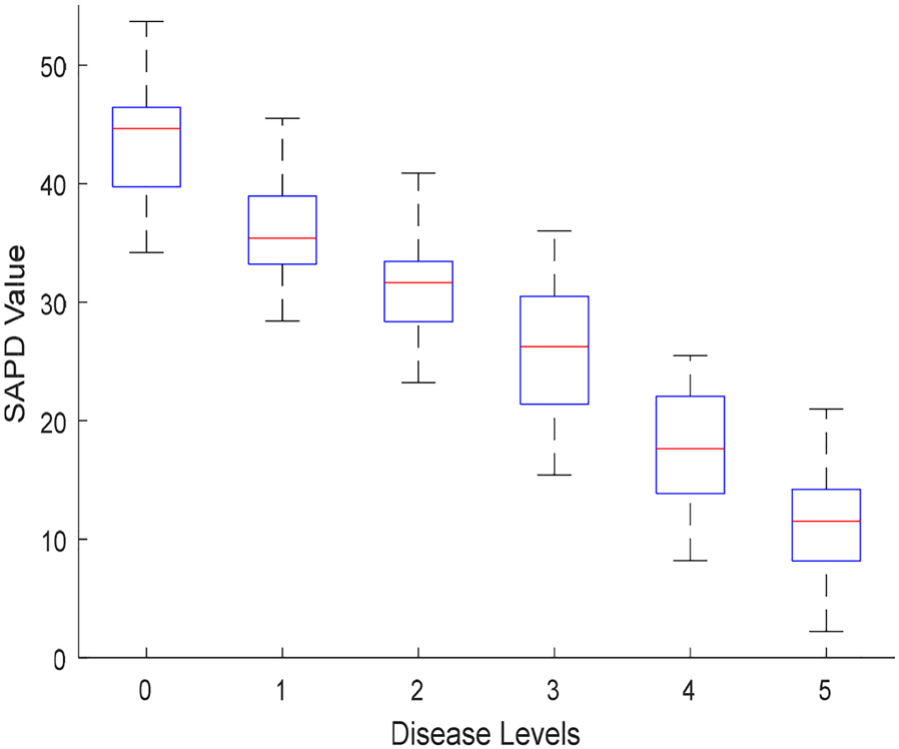
SPAD value of rice leaves under different disease levels. The top and bottom black lines represent the maximum and minimum SPAD values, respectively. The red line represents the average SPAD value. The number of rice leaves under disease levels 0 to 5 is 200, 170, 160, 200, 140, and 130, respectively

The SPAD value varies in the chlorophyll content, ranging from 0 to 60, and the change of the SPAD value under different disease levels is shown in Fig. 8.

It can be seen from Fig. 8 that the average SPAD value of diseased rice leaves shows a downward trend. This finding is consistent with previous research, which found that chlorophyll content drops rapidly when plants are stressed and during leaf senescence [58]. In the early stage of infection, a small number of bacteria cannot cause extensive damage to leaves, and the diffusion rate is slow, so there is minor change in the leaf chlorophyll content in the infected area [59]. The chlorophyll content of rice leaves at level 0 is the highest, with the maximum value of 53.7 and an average value of 44.65. The SPAD value is mainly affected by the leaf structure and the cell structure of rice leaves, both of which are damaged after the bacterial blight infection. As the disease further develops, the average SPAD value gradually decreases to 11.5 (when the disease level reaches Level 5).

### Correlation analysis between VIs and SPAD value

**Fig. 9:**
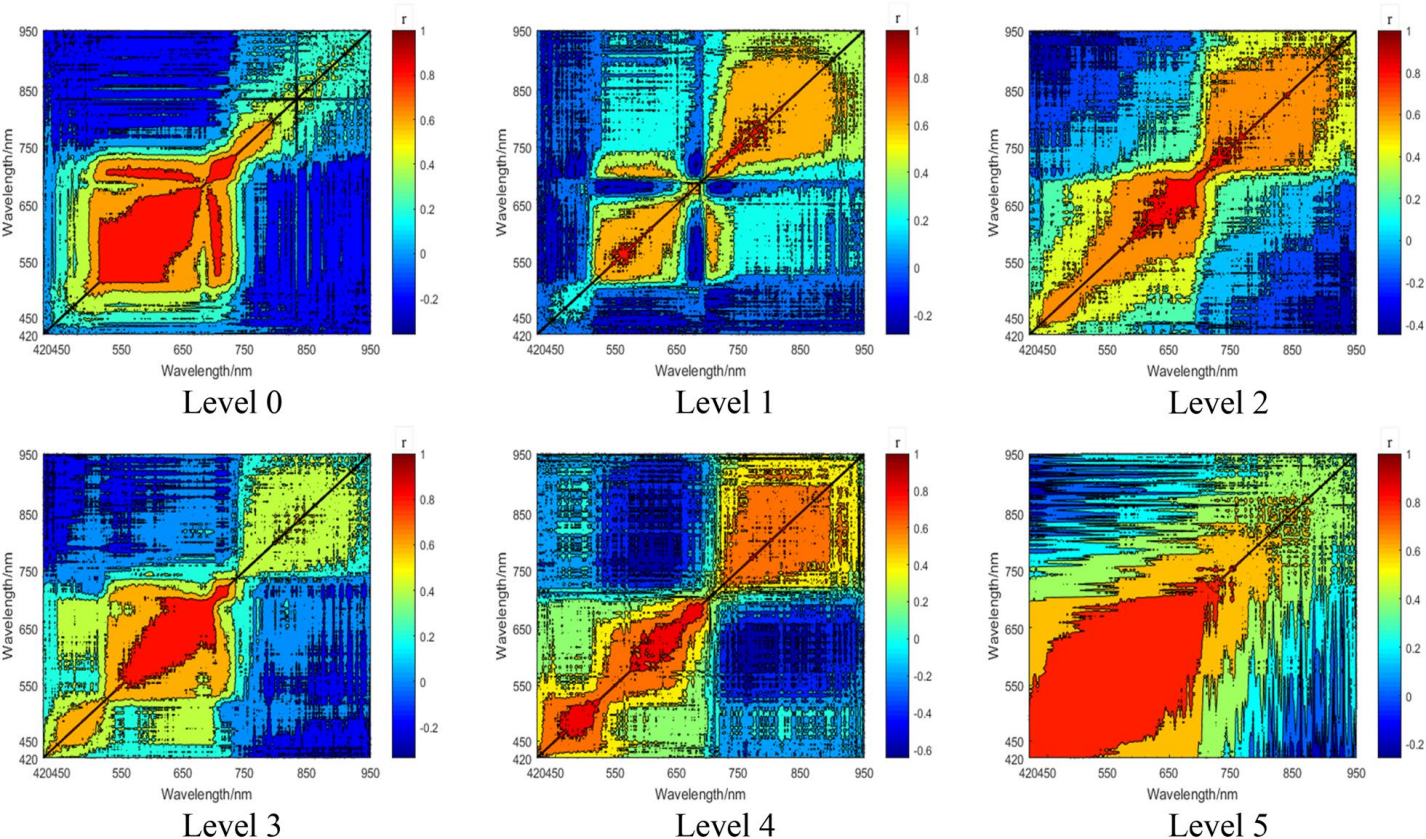
Contour maps of the coefficients of determination for the relationship between hyperspectral bands and SPAD values under six disease levels. In the color bar, the color changed with the correlation, from 0 to 1, meaning the stronger the positive correlation between the SPAD value and the spectral band. And from 0 to − 1, meaning the stronger the negative correlation between the SPAD value and the spectral band

When using current VIs to predict the SPAD value, it is noted that some indices will become saturated with the change of the measured parameters (e.g., the chlorophyll content). As a result, these indices would become less sensitive to changes of SPAD values under different disease levels and eventually achieve poor prediction accuracy. In order to determine the hyperspectral bands that are strongly correlated with the SPAD value of rice leaves under the bacterial blight disease stress, the contour maps of the coefficients of determination for the relationship between spectra and SPAD values under six different disease levels were analyzed (Fig. 9).

Figure 9 shows that the SPAD value is correlated with certain hyperspectral bands under different disease levels. For instance, for the disease level 0, hyperspectral bands from 500 to 650 nm show a strong correlation with the SPAD value. For the disease level 3, a strong correlation appears at bands that range from 560 to 720 nm. Lastly, for the disease level 5, bands from 420 to 700 are positively correlated with the SPAD value. Therefore, it can be concluded that current VIs cannot accurately predict the SPAD value of rice leaves under the bacterial blight disease stress, due to the reason that the selection of sensitive bands is usually fixed and limited. For example, NDVI only concerns the wavelengths at 670 and 800 nm. Under such circumstances, NDVI may become less sensitive to the disease level 4, because the sensitive bands for this disease level correspond to 480–550 nm and 620–680 nm according to our correlation analysis. The result of the statistical analysis presented in Table 4 also supports this finding. Among the 17 VIs, including the SFDI, 9 VIs have a correlation coefficient with the SPAD value exceeding 0.5000, and 6 of them reach over 0.7000. The correlation between the SFDI and the SPAD value is the strongest, reaching the correlation coefficient of 0.8263.

**Table 4:** Correlations between SPAD value and VIs

VIs	Correlation coefficient
SFDI	0.8263**
MSAVI	0.8024**
RVI	0.7947**
VARI_red_	0.7235**
NPCI	0.6426*
NDVI	0.5545**
SAVI	0.4989*
GNDVI	0.4559*
VOG_3_	0.3125**
VOG_1_	− 0.2692**
NDI	− 0.3752**
VOG_2_	− 0.4041**
PSRI	− 0.4591*
PRI	− 0.4779**
VARI_green_	− 0.5852**
MTCI	− 0.7541**
MCARI	− 0.7578**

* and ** indicate that correlations are significant at confidence levels of 0.05 and 0.01, respectively

### Model evaluation with selected VIs

SFDI, MSAVI, RVI, VARI_red_, MTCI, and MCARI were selected for regression analysis. VIs under different disease levels were used as input variables, and the desired output variable is the SPAD value. Datasets of hyperspectral images and their corresponding SPAD values were divided into training and testing sets in an 8:2 ratio and a K-fold cross validation approach is adopted (K = 5). The regression models were established based on four machine learning models, including decision tree (DT), partial least square regression (PLSR), support vector regression (SVR), and back propagation neural network (BPNN). The prediction performance of each model built with selected VIs was presented in Table 5.

**Table 5:** Performance of prediction models built with different VIs

VIs	Model	Training set			Test set		
		R^2^	RMSE	RE/%	R^2^	RMSE	RE/%
MSAVI	DT	0.8153	4.2358	9.3182	0.7916	4.7874	9.5617
	PLSR	0.8019	5.2545	10.2113	0.7711	5.3593	10.3156
	SVR	0.8553	4.0548	8.3935	0.8355	4.5187	9.3219
	BPNN	0.8437	3.2254	8.7417	0.8322	3.3290	10.8533
MCARI	DT	0.7215	8.3541	14.0523	0.7006	9.2147	15.3319
	PLSR	0.6853	7.4097	11.0561	0.6631	10.2416	12.1102
	SVR	0.7783	6.8345	9.7764	0.7547	10.9724	9.1542
	BPNN	0.7512	6.7714	10.2314	0.7431	9.2433	10.2011
MTCI	DT	0.5839	10.9318	20.2176	0.5581	18.5998	20.3154
	PLSR	0.6337	13.4315	17.7154	0.6255	14.3392	18.6833
	SVR	0.6239	8.3549	13.1171	0.6213	10.4582	14.6914
	BPNN	0.6617	7.9018	12.1272	0.6571	9.8851	13.2387
RVI	DT	0.5311	12.2155	19.2513	0.4924	13.5217	19.7315
	PLSR	0.5442	11.5125	18.2651	0.5351	12.3652	18.9113
	SVR	0.5537	9.2254	13.7114	0.5463	10.3290	13.8151
	BPNN	0.5329	8.7592	14.2615	0.5154	9.3651	14.3216
VARI_red_	DT	0.7419	9.8263	10.2344	0.7224	10.2355	12.9371
	PLSR	0.7133	12.3652	14.2615	0.7062	13.6239	16.3117
	SVR	0.7939	11.9217	10.0200	0.7819	12.9759	13.8592
	BPNN	0.7785	8.8251	9.3154	0.7435	10.9472	9.8138
SFDI	DT	0.8413	4.5163	10.5127	0.8387	4.7184	10.6479
	PLSR	0.8516	3.8715	9.8435	0.8479	4.5526	9.9316
	SVR	0.8874	3.5124	7.7451	**0.8752**	**3.7715**	**7.8614**
	BPNN	0.8759	3.3152	8.3218	0.8679	3.6780	8.6153

The bold values highlight the best performance

As shown in Table 5, all regression models built with SFDI achieve the optimal performance. For the test set, the result of R^2^ reaches over 0.8387, while the results of RMSE and RE are below 4.7184 and 10.6479%, respectively. In particular, the regression model based on SVR is the optimal one, reaching R^2^, RMSE, and RE at 0.8752, 3.7715, and 7.8614%, respectively.

**Fig. 10:**
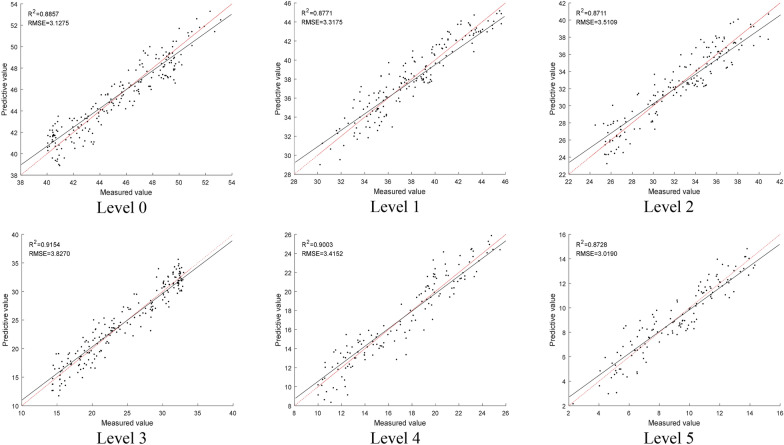
Prediction results of the SVR model built with the SFDI. The red line represents x = y (R^2^ = 1), and the black line indicates the fitting performance

Therefore, by using the SVR model built with the SFDI, the SPAD value can be predicted more accurately and robustly. The prediction performance of the model established by SVR and SFDI under six disease levels is shown in Fig. 10. The R^2^ indicates the distance of the measured data from the regression line, which gives the overall effect of the regression model. The closer R^2^ is to 1 and the smaller the RMSE, and the closer the slope of the regression equation is to 1, the better the model fitting ability and prediction ability.

It can be seen from Fig. 10 that the SVR model built with the proposed SFDI demonstrates outstanding stability and accuracy in predicting the SPAD value. The prediction performance of SPAD value under the disease level 3 is the best, the determination coefficient R^2^ reaches the highest at 0.9154, and the RMSE is 3.8270. Overall, the values of R^2^ and RMSE for all 6 disease levels only have a minor difference. As a result, the SFDI can overcome the saturation problem of current VIs in predicting the SPAD value of the rice leaves under the bacterial blight disease stress by providing a more robust result.

## Discussion

### Discussion about sensitive bands and prediction performance

To determine the SPAD value of infected rice leaves, VIs established with hyperspectral information have been applied. As a non-destructive method for qualitative and quantitative analysis, the hyperspectral technology plays an important role. Bacterial blight would accelerate the process of chlorophyll degradation and leaf structure destruction, causing a dramatic change in the hyperspectral information, which has been explained in previous studies [26, 60]. In this work, the SFDI has been proven to be a robust and accurate index for predicting the SPAD value of infected rice leaves because it contains more hyperspectral information and can explain the irregularity of hyperspectral curves. Through radius iteration, the minor changes of reflectance curves between any two adjacent hyperspectral wavelengths can be detected by the SFDI.

In the visible light region, especially the green light region, the reflectance is mainly affected by leaf pigments [61]. At the early stage of the bacterial blight disease, the chlorophyll content in the rice leaves gradually decreases, and the color of leaves changes from green to yellow. The hyperspectral curve shows a green peak at around 550 nm, where leaves absorb green light at the highest rate, leading to the lowest reflectance of green light. Meanwhile, there is a red valley at around 680 nm, where the leaves absorb the most red-light, resulting in the lowest red-light reflectance. As the BB disease progresses, chloroplasts suffer severe damage. The SPAD value decreases, and the reflectance of green and red-light regions increases [59]. The green peak and red valley disappear at this time. It is worth mentioning that the reflectance of the red-light region rises faster than that of the green light region, and the original peak-valley curve gradually becomes a parabola.

It is noted that current vegetation indices only adopt few bands selected. For instance, MSAVI is composed of near-infrared and red-light regions [48], while NDVI consists of wavelengths at 670 and 800 nm [49]. Wavelengths at 531 and 570 nm are used to establish PRI [49]. According to our analysis, the combination of different bands may have an influence on the prediction accuracy, this is consistent with the findings of Zhang [24]. On the one hand, VIs composed of more wavelengths, such as SFDI, MSAVI, and RVI, would be more strongly correlated with the SPAD value under the bacterial blight disease stress. Due to the adequate inclusion of hyperspectral information in the red-light and near-infrared regions, the correlation coefficients of these indices reach over 0.7900 with the SPAD value. On the other hand, the rest VIs that contain less hyperspectral information are all weakly correlated with the SPAD value.

### Discussion about sensitive bands under different disease levels

We also find that the SPAD value of rice leaves under different disease levels is sensitive to different bands, which corresponds to the findings of the literature [60]. According to Yu et al. [62], leaf reflectance is mainly affected by the pigment, cell structure, and leaf water content in the leaf, while in the visible light waveband (350–720 nm), leaf reflectance is most affected by chlorophyll. From 420 to 680 nm, a downward trend in the light absorption rate is shown, especially in the red valley region where the chlorophyll light absorption ability is stronger and the reflectance shows an upward trend, the same as in Jin’s research results [63]. A peak and a valley appear at around 550 nm and 680 nm, respectively. Meanwhile, the hyperspectral reflectance increases dramatically between 650 and 700 nm. It means that the current VIs may achieve poor accuracy. For instance, NPCI (with fixed wavelengths at 430 and 680 nm) may fail to predict the SPAD value of rice leaves under the disease level 1, because the sensitive bands for this level are 550–570 nm and 710–740 nm. Fortunately, the proposed index, SFDI, takes advantage of adopting a period of continuous bands ranging from 420 to 950 nm, which covers adequate hyperspectral information.

### Discussion about sensitive bands and various stresses

There are many causes for the change of the hyperspectral reflectance, such as insect, drought, and vegetation deficiency stresses [29, 58, 61]. Plenty of studies have provided a solid foundation for the effective application of spectrum in rice disease detection and the prediction of biochemical parameters of rice leaves under various stresses [7, 24, 26], while the sensitive bands are different under various stresses. All these stresses may have potential influences on the hyperspectral characteristics, thus affecting the accuracy of the SPAD prediction. The development of new indices for predicting the SPAD value considering various stresses and wavelengths would be a very interesting topic to explore in future studies. Furthermore, a stable index is urgently needed to quantitatively analyze changes of the rice leaf spectrum under different stresses, as well as to predict biochemical parameters and detect diseases under different stresses.

## Conclusion

Periodically estimating the chlorophyll content is essential to monitor the general health of rice. The ultimate objective is to maximize the crop yield and reduce losses caused by the bacterial blight disease spreading among crops. Current VIs (e.g., MSAVI, MCARI, MTCI, etc.) are established by considering only the sensitive (e.g., 670 and 450 nm) and anti-intervention wavelengths (e.g., 700 and 550 nm). However, the combination of selected bands cannot fully reflect the minor changes of the chlorophyll content in plants under disease stress. Because the ignorance of certain wavelengths may lead to the loss of sensitive information, thereby decreasing the prediction accuracy. Besides, it is found that the SPAD value of rice leaves under different disease levels is sensitive to different wavelengths. In this work, to overcome the limitations of current VIs, a robust index, SFDI, is proposed by computing the fractal dimension of a hyperspectral curve ranging from 420 to 950 nm. The experimental result demonstrates that the SFDI shows a stronger positive correlation with the SPAD value than current ones. We further build a SPAD prediction model by combining the SFDI with various machine learning models. The comparison result over the testing dataset shows that the prediction model based on SVR and SFDI achieves the best prediction performance, reaching R^2^, RMSE, and RE at 0.8752, 3.7715, and 7.8614%, respectively.

In conclusion, it is recommended to consider to use this newly-proposed index, SFDI, to robustly and accurately predict the SPAD value of rice leaves under the bacterial blight stress with the hyperspectral information ranging from 420 to 950 nm. Further exploration of our study may focus on testing the SFDI over other plants to verify its applicability and usefulness.

## Supplementary Information

**Additional file 1: Figure S1.** **a** Cultivation of the pathogenic bacteria *Xanthomonas oryzae pv. Oryzae*. **b** Infection of the bacterial blight. **Figure S2.** **a** Original reflectance curve. **b** SG smoothed reflectance curve. **Figure S3.** Measurement of SPAD using the SPAD-502 meter. **Table S1. **Main components of the imaging system and parameter settings. **Table S2. **Imaging parameters used in this experiment. **Table S3. **Specifications of the SPAD-502 meter.

## Data Availability

The remotely sensed and field sampling data used in this study is available from the corresponding author on reasonable request.

## Abbreviations

BB: Bacterial blight
SPAD: Soil–plant analysis development
VI: Vegetation index
DT: Decision tree
PLSR: Partial least square regressions
SVR: Support vector regression
BPNN: Back propagation neural network
SFDI: Spectral fractal dimension index
HSI: Hyperspectral image
ROI: Region of interest
SG: Savitzky–Golay
GNDVI: Green normalized difference vegetation index
MCARI: Modified chlorophyll absorption in reflective index
VOG: Vogelmann index
PSRI: Plant senescence reflectance index
MSAVI: Modified soil-adjusted vegetation index
NDVI: Normalized difference vegetation index
PRI: Photochemical reflectance index
NPCI: Normalized pigment chlorophyll index
MTCI: MERIS terrestrial chlorophyll index
RVI: Ratio vegetation index
NDI: Normalized vegetation index
SAVI: Soil-adjusted vegetation index
VARI: Visible atmospherically resistant index
R^2^: Coefficient of determination
RMSE: Root mean square error
RE: Relative error
MSE: Mean square error
