# Supplementary material for: Applying spectral fractal dimension index to predict the SPAD value of rice leaves under bacterial blight disease stress
Source: Plant Methods. 2022 May 18;18:67. doi: 10.1186/s13007-022-00898-8 (PMC9118648; doi:10.1186/s13007-022-00898-8)
Supplement: Supplementary file 1 — Additional file 1: Figure S1. a Cultivation of the pathogenic bacteria Xanthomonas oryzae pv. Oryzae. b Infection of the bacterial blight. Figure S2. a Original reflectance curve. b SG smoothed reflectance curve. Figure S3. Measurement of SPAD using the SPAD-502 meter. Table S1. Main components of the imaging system and parameter settings. Table S2. Imaging parameters used in this experiment. Table S3. Specifications of the SPAD-502 meter. [file 13007_2022_898_MOESM1_ESM.pdf]

Additional file 1

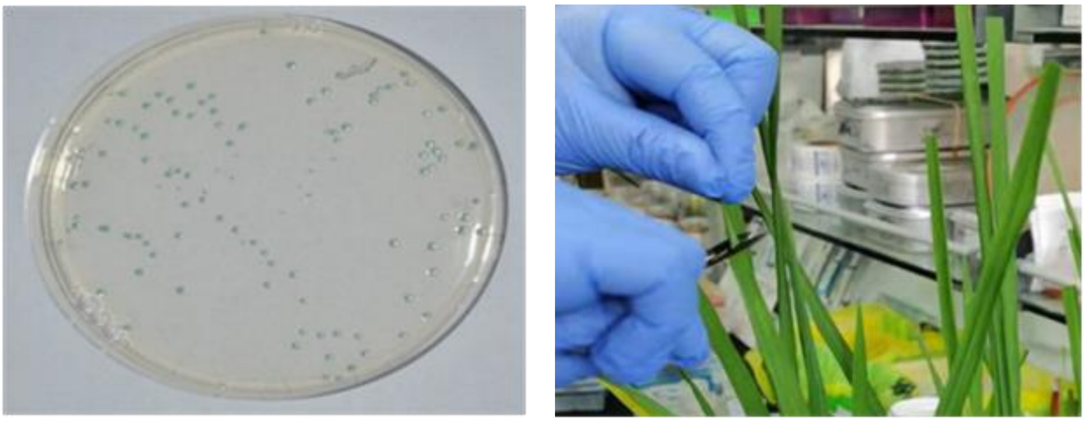

Fig. S1. (a) Cultivation of the pathogenic bacteria *Xanthomonas oryzae pv. Oryzae*. **(b)** Infection of the bacterial blight. **Note: We used a pair of scissors with dipped pathogenic bacteria to cut off the top leaf of healthy rice for the purpose of infection. The infection was performed when the 5th leaf of rice started emerging.**

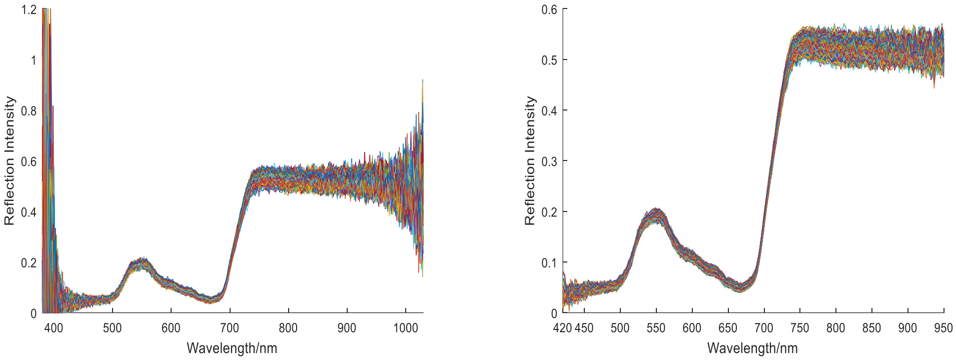

Fig. S2. (a) Original reflectance curve. **(b)** SG smoothed reflectance curve.

In Fig. S2 (a), it is noted that the reflectance at 378-420 nm and 950-1033 nm bands contain much noise. Meanwhile, for reducing the computational costs, both ends are cut off and bands ranging from 420-950 nm are maintained for further analysis. In Fig. S2 (b), we adopted the SG smoothing filter. This filter calculates the average value of a set of sample raw spectra following a moving smoothing window. After the smoothing operation, the reflectance was maintained at the interval from 0 to 0.6, which is consistent with the original spectra.

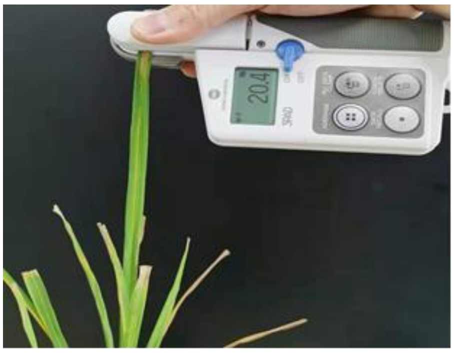

Fig. S3. Measurement of SPAD using the SPAD-502 meter.

The sample leaf was placed between the 2 heads of the meter to measure the SPAD value. The SPAD value for each leaf was measured three times, and the average value was calculated for record.

Table S1. Main components of the imaging system and parameter settings

Parameters	Value (unit)
Spectral camera	Raptor EM285
Spectral range	378~1033 nm
Dispersion	97.5nm/mm
Spectral resolution	2.8 nm
Image size	6.15mm*14.2mm
Spatial resolution	Spot diameter <9 um
Aberration	Halo <1.5 um, trapezoid <1 um
Aperture	F/2.4.
Light input	21V/200W halogen light
Efficient	50%

Table S2. Imaging parameters used in this experiment

Parameters	Values
Lens focal length	35 mm
Object distance	27 cm
Light source	130 lx
Exposure time	10 ms

Table S3. Specifications of the SPAD-502 meter

Attribute	Value
Measurement area	2mm × 3mm
Measurement range	- 9.9 ~ 199.9 SPAD unit
Measurement duration	2 seconds
Accuracy	± 1.0 SPAD unit
